# Cerebrospinal fluid neurofilament heavy protein levels associated with domain-specific cognitive decline in Alzheimer’s disease

**DOI:** 10.3389/fnagi.2026.1826215

**Published:** 2026-10-22

**Authors:** Jianjing Zhang, Xiwu Wang, Lingkai Yang, Zhaoting Lv, Suilin Jia, Teng Ye

**Affiliations:** 1 Department of Psychiatry, Wenzhou Seventh People’s Hospital, Wenzhou, Zhejiang, China; 2 Department of Psychiatry, Tongde Hospital of Zhejiang Province, Hangzhou, Zhejiang, China; 3 Department of Medical Ultrasound, Wenzhou Key Laboratory of Interventional Ultrasound for Intelligent Healthcare and Clinical Translation, the First Affiliated Hospital of Wenzhou Medical University, Wenzhou, Zhejiang, China

**Keywords:** Alzheimer’s disease, cerebrospinal fluid, cognitive decline, disease progression, neurofilament heavy chain

## Abstract

**Background:**

Neurofilament proteins are fluid biomarkers of neuroaxonal damage in various neurological diseases. However, it remains to be elucidated whether CSF neurofilament heavy (NfH) protein levels correlate with cognitive decline and progression in Alzheimer’s disease (AD).

**Methods:**

This study included 165 cognitively unimpaired (CU) participants, 385 participants with mild cognitive impairment (MCI), and 128 participants with AD. To examine correlations between CSF NfH levels and baseline cognitive measures, Pearson’s correlation was used. Linear mixed-effects models were fitted to assess the relationship between elevated CSF NfH levels and longitudinal cognitive decline in the CU, MCI and AD groups. Kaplan–Meier survival analyses and Cox proportional hazards models were used to assess the clinical progression in the CU and MCI groups separately.

**Results:**

In the MCI group, higher CSF NfH levels were significantly associated with poorer performance across global measures (MMSE, CDR-SB, ADAS-Cog-13, and RAVLT immediate recall; *p* < 0.001) and three cognitive domains (such as memory, language, and executive function), in both cross-sectional and longitudinal analyses. In contrast, the significant associations in the CU and AD groups were observed for a subset of cognitive tests and domains. Kaplan–Meier survival analyses showed that the high CSF NfH group had higher progression rates than the low group in both the CU and MCI groups. In the Cox proportional hazards models, baseline CSF NfH (continuous) remained a significant predictor of clinical progression.

**Discussion:**

Higher CSF NfH levels were associated with faster cognitive decline and clinical progression in CU and MCI groups but not in participants with AD, suggesting its potential as a biomarker for tracking early disease progression and cognitive impairment.

## Introduction

Alzheimer’s disease (AD) is a progressive neurodegenerative disorder which begins with memory impairment and cognitive decline, and progresses to affect behavior, speech, and motor function (Scheltens et al., 2021). A growing body of evidence indicates that cerebrospinal fluid (CSF) biomarkers of neurodegeneration demonstrate fair-to-good performance in predicting cognitive decline, dementia, and its progression (Olsson et al., 2016; Dage et al., 2023).

Neurofilament proteins, as major cytoskeletal components of myelinated axons, are composed of a heteropolymer core of neurofilament heavy (NfH), medium (NfM), and light (NfL) chains (de Jong et al., 2007), with possible incorporation of α-internexin or peripherin (Yuan et al., 2017; Wang et al., 2022). Upon axonal damage, they are released into extracellular fluids such as cerebrospinal fluid (CSF) and blood. Given the established role of neurofilaments as biomarkers for disease progression, prognosis, and treatment response in neurodegeneration (Kuhle et al., 2011; Khalil et al., 2018), and especially since NfL has been extensively characterized in AD (Zetterberg et al., 2016; Ashton et al., 2019; Olsson et al., 2019), the patterns of association between NfH and multiple cognitive domains in AD remain comparatively unclear.

In this study, our primary goal was to investigate whether baseline CSF NfH levels are correlated to global cognition, subsequent cognitive decline, as well as the clinical progression of AD. Furthermore, we examined associations between NfH levels and specific cognitive domains, including memory, language, executive function, and visuospatial function.

## Methods

### Participants and data sources

Data used in this study were downloaded from the Alzheimer's Disease Neuroimaging Initiative (ADNI) database on March 20, 2025. In the current study, we focused on the 678 participants who were classified as CU (*n* = 165), MCI (*n* = 385), and AD (*n* = 128). All of them had data on demographics and other clinical variables, including baseline age, educational years, sex, APOE4 status (non-carriers vs. carriers) and cognitive status (CU, MCI and AD). Individuals were included in this analysis if they had baseline cerebrospinal fluid (CSF) neurofilament heavy chain (NfH) protein levels, and baseline and at least one follow-up assessment of Mini-Mental State Examination (MMSE) in the next 10 years (Table 1). The sample selection showed in Supplementary Figure S1. Supplementary Table S1 listed the sample size of follow-up visits in the supplement. Specific inclusion and exclusion criteria were described previously (Petersen et al., 2010; Aisen et al., 2024) and are also found on the website.^1^ Briefly, CU participants had an MMSE score between 24 and 30, and a CDR score of 0.Participants meeting the criteria for MCI had an MMSE score of 24 or higher, a CDR score of 0.5, objective memory impairment (based on delayed recall scores from Logical Memory II of the Wechsler Memory Scale-Revised), and an absence of dementia. Participants with AD had a MMSE score between 20 and 26, a CDR score of 0.5 or 1, meeting the diagnostic criteria of probable AD (McKhann et al., 1984; Aisen et al., 2024). Each ADNI site obtained local Institutional Review Board protocol approval, and all participants and/or their authorized representatives gave written informed consent to participate in the study (Aisen et al., 2024).

**Table 1:** Baseline demographical and clinical characteristics.

Characteristic	Overall	CU	MCI	AD	*p*-value^b^
	*N* = 678^a^	*N* = 165^a^	*N* = 385^a^	*N* = 128^a^	
Age, y	74 (68, 78)	73 (71, 78)	73 (67, 77)	76 (71, 81)	<0.001
Education, y	16 (14, 18)	16 (14, 18)	16 (14, 18)	16 (13, 18)	0.050
Female, (%)	290 (43%)	76 (46%)	163 (42%)	51 (40%)	0.5
APOE ε4 carriers, *n* (%)	334 (49%)	40 (24%)	202 (52%)	92 (72%)	<0.001
Length of follow-up, years	3.85 (2.67)	5.2 (2.99)	4.03 (2.46)	1.56 (0.62)	NA
CSF NfH, RFU	258 (222, 301)	238 (207, 270)	254 (222, 297)	299 (258, 328)	<0.001
MMSE score	28 (26, 29)	29 (29, 30)	28 (26, 29)	23 (22, 25)	<0.001
ADAS-Cog 13	16 (10, 23)	9 (7, 13)	17 (11, 21)	29 (24, 34)	<0.001
* Missing, n*	5	0	1	4	
CDRSB score	1.50 (0.50, 2.50)	0.00 (0.00, 0.00)	1.50 (1.00, 2.00)	4.50 (3.50, 5.50)	<0.001
RAVLT immediate recall	33 (25, 43)	44 (37, 52)	33 (27, 40)	22 (18, 26)	<0.001
* Missing, n*	2	0	0	2	
Memory composite score	0.20 (−0.51, 0.82)	1.00 (0.62, 1.52)	0.13 (−0.30, 0.66)	−1.01 (−1.47, −0.61)	<0.001
Executive composite score	0.19 (−0.47, 0.88)	0.74 (0.34, 1.51)	0.22 (−0.32, 0.88)	−0.80 (−1.48, −0.30)	<0.001
Visuospatial composite score	−0.08 (−0.66, 0.74)	0.74 (−0.08, 0.74)	−0.08 (−0.55, 0.74)	−0.74 (−1.36, −0.08)	<0.001
Language composite score	0.25 (−0.35, 0.82)	0.79 (0.36, 1.33)	0.23 (−0.22, 0.77)	−0.56 (−1.16, −0.14)	<0.001
Aβ status: positive, *n* (%)	445 (66%)	63 (38%)	262 (68%)	120 (94%)	<0.001

a Median (Q1, Q3); *n* (%).

b Differences of baseline demographic and clinical variables, and CSF biomarkers between three diagnostic groups were analyzed by the Kruskal-Wallis rank sum test and Pearson's Chi-squared tests depending on types of variables concerned.

AD, Alzheimer’s Disease; ADAS-Cog-13, Alzheimer’s Disease Assessment Scale, 13-item cognitive subscale; APOE, apolipoprotein E; CDR-SB, Clinical Dementia Rating Sum of Boxes; Aβ, amyloid-β; CSF, cerebrospinal fluid; CU, cognitively unimpaired; MCI, mild cognitively impaired; MMSE, Mini-Mental State Examination; NfH, neurofilament heavy; RFU, relative fluorescence units; RAVLT, Rey auditory verbal learning test.

*ADNI*: Data used in the preparation of this article were obtained from the ADNI database (adni.loni.usc.edu). The ADNI was launched in 2003 as a public-private partnership, led by Principal Investigator Michael W. Weiner, MD. The primary goal of ADNI has been to test whether serial magnetic resonance imaging (MRI), positron emission tomography (PET), other biological markers, and clinical and neuropsychological assessment can be combined to measure the progression of mild cognitive impairment (MCI) and early Alzheimer’s disease (AD). For up-to-date information, see http://www.adni-info.org.

### Neuropsychological assessments

We leveraged four primary cognitive assessments in this study. The MMSE score, a brief screening test of global cognition, ranges from 0 to 30, with lower scores reflecting worse cognitive performance (Folstein et al., 1975). The Clinical Dementia Rating Sum of Boxes (CDR-SB) ranges from 0 to 18, with higher scores indicating more severe impairment (Morris, 1993). The Alzheimer’s Disease Assessment Scale-Cognitive Subscale 13 (ADAS-Cog-13), an examination of 13 aspects of cognition, ranges from 0 to 85, with lower scores representing better cognition (Rosen et al., 1984). Verbal episodic memory was assessed using the Rey Auditory Verbal Learning Test (RAVLT), with higher scores donating better performance (Schmidt, 1996; Sundermann et al., 2016). In this study, MMSE, CDR‑SB, ADAS‑Cog‑13, and RAVLT were analyzed as raw scores (untransformed), consistent with previous ADNI study (Petersen et al., 2010).

The four composite scores for memory (ADNI‑MEM), executive function (ADNI‑EF), language (ADNI‑LAN), and visuospatial functioning (ADNI‑VS) were developed and calculated from the ADNI neuropsychological battery (Petersen et al., 2010) using item response theory (IRT) methods, as reported previously (Crane et al., 2012; Gibbons et al., 2012; Choi et al., 2020). We used the composite scores that were pre‑computed and provided in the ADNI dataset, generated using the methods described in the previous papers.

### APOE ε4 status

Blood samples were collected from participants for at the screening visit for APOE genotyping (Petersen et al., 2010). APOE ε4 status was categorized into two groups: non-carriers (subjects without an ε4 allele) and carriers (subjects with one or two ε4 alleles).

### Cerebrospinal fluid biomarker measurements

The CSF samples were collected and stored as previously described (Harvey et al., 2024; Shaw et al., 2024). All processing, storage, shipment, and handling of CSF samples were performed on dry ice, with long-term storage at −80 °C in the ADNI Biofluid Biobank at the University of Pennsylvania (UPENN) (Shaw et al., 2024). The CSF Aβ42 levels were measured using the Elecsys® Aβ42 CSF immunoassay at UPENN (Hansson et al., 2018) in accordance with the manufacturer's instructions (Shaw et al., 2016; Lifke et al., 2019), and described previously (Bittner et al., 2016; Hansson et al., 2018). The measuring range of the Elecsys Aβ42 was from 200 to 1700 pg/mL. The cutoff for Aβ status was defined as follows: baseline CSF Aβ42 level < 1098 pg/mL was considered Aβ positive, and a level ≥ 1098 pg/mL was considered Aβ negative (Schindler et al., 2018).

The levels of CSF NfH and NfL were measured using the SomaScan 7 K platform by the Neurogenomics and Informatics Center at Washington University, normalized by SomaLogic and reported in relative fluorescence units (RFU). The details regarding quality control and data generation can be found in the previous documents (SomaLogic, 2021; Western et al., 2024).

### Statistical analysis

Differences in demographic, clinical, and biomarker variables among diagnostic groups (CU, MCI, and AD) were assessed using the Kruskal-Wallis rank sum test for continuous variables and Chi-squared tests for categorical variables.

To detect the correlational associations between CSF NfH levels and baseline cognitive measures, Pearson’s correlation analysis was applied. For subjects with longitudinal data, linear mixed-effects models were used to investigate the relationship between baseline CSF NfH levels and longitudinal declines in cognitive performance. The models included baseline NfH levels, time (years of follow-up), and their interaction as fixed effects, along with random intercepts and random slopes for time, adjusting for age, sex, years of education, APOE ε4 status, and Aβ status. To detect group differences, these models were fitted for each diagnostic group separately. Several core model assumptions were assessed, including the normality of residuals and random effects. Most of the tested models did not substantially violate these assumptions; however, minor deviations from normality were detected for CDR-SB in the CU group. Nevertheless, linear mixed-effects models are generally robust to violations of distributional assumptions (Schielzeth et al., 2020). The false discovery rate (FDR) correction was applied to all linear mixed-effects models (Supplementary Table S8).

Kaplan–Meier survival analysis was conducted to assess the relationship between baseline CSF NfH (as a categorical variable) and clinical progression (from CU to MCI/AD and from MCI to AD). In this analysis, NfH was dichotomized into low and high subgroups based on group-specific median levels (238.4 RFU for CU, 254.4 RFU for MCI). In contrast, baseline CSF NfH was included as a continuous variable in the multivariable Cox proportional hazards models.

All data analyses in this paper were performed using the R statistical software (version 4.5.1) (R Core Team, 2019). All linear mixed-effects models were fitted using the lme4 package in R. The significance level was set at *p* < 0.05.

### Sensitivity analysis

To further assess whether the associations were specific to NfH or NfL, we repeated the linear mixed‑effects analyses for CSF NfL in each diagnostic group adjusted for covariates.

Given the differences in amyloid status between CU ang MCI groups, linear mixed-effects models were performed to test the three‑way interaction effects of NfH, Aβ status and follow-up time in CU and MCI separately. Furthermore, Aβ-stratified analyses were conducted to estimate the association between baseline CSF NfH and cognitive decline. Models were adjusted for age, sex, years of education, and APOE ε4 status.

## Results

### Baseline demographics and clinical characteristics

In this analysis, a total of 678 participants were tincluded: 165 with CU (mean [SD] age, 74 [6] years; 76 female [46%]), 385 with MCI (mean [SD] age, 72 [7] years; 163 female [42%]), and 128 with AD (mean [SD] age, 75 [9] years; 51 female [40%]). The average follow-up duration was 3.85 (SD = 2.67) years. The participant characteristics are presented in Table 1. The number of participants at each follow-up time point is summarized in Supplementary Table S1.

Compared with participants in the CU and MCI groups, individuals with AD exhibited fewer education years, higher frequency of APOE ε4 carriage, lower MMSE and RAVLT immediate recall scores, and higher CDR-SB, ADAS-Cog-13 scores, and CSF NfH levels. Significant differences were also observed across multiple cognitive domains (memory, executive function, language and visuospatial function), and amyloid status at baseline (Table 1).

### Cross-sectional relationship between CSF NfH levels and cognition

To examine the cross-sectional associations between the CSF NfH levels and multiple cognitive measures, including MMSE, CDR-SB, ADAS-Cog-13 and RAVLT immediate recall scores, we performed Pearson’s correlation analyses separately for participants in the CU, MCI and AD groups (Figure 1).

**Figure 1:**
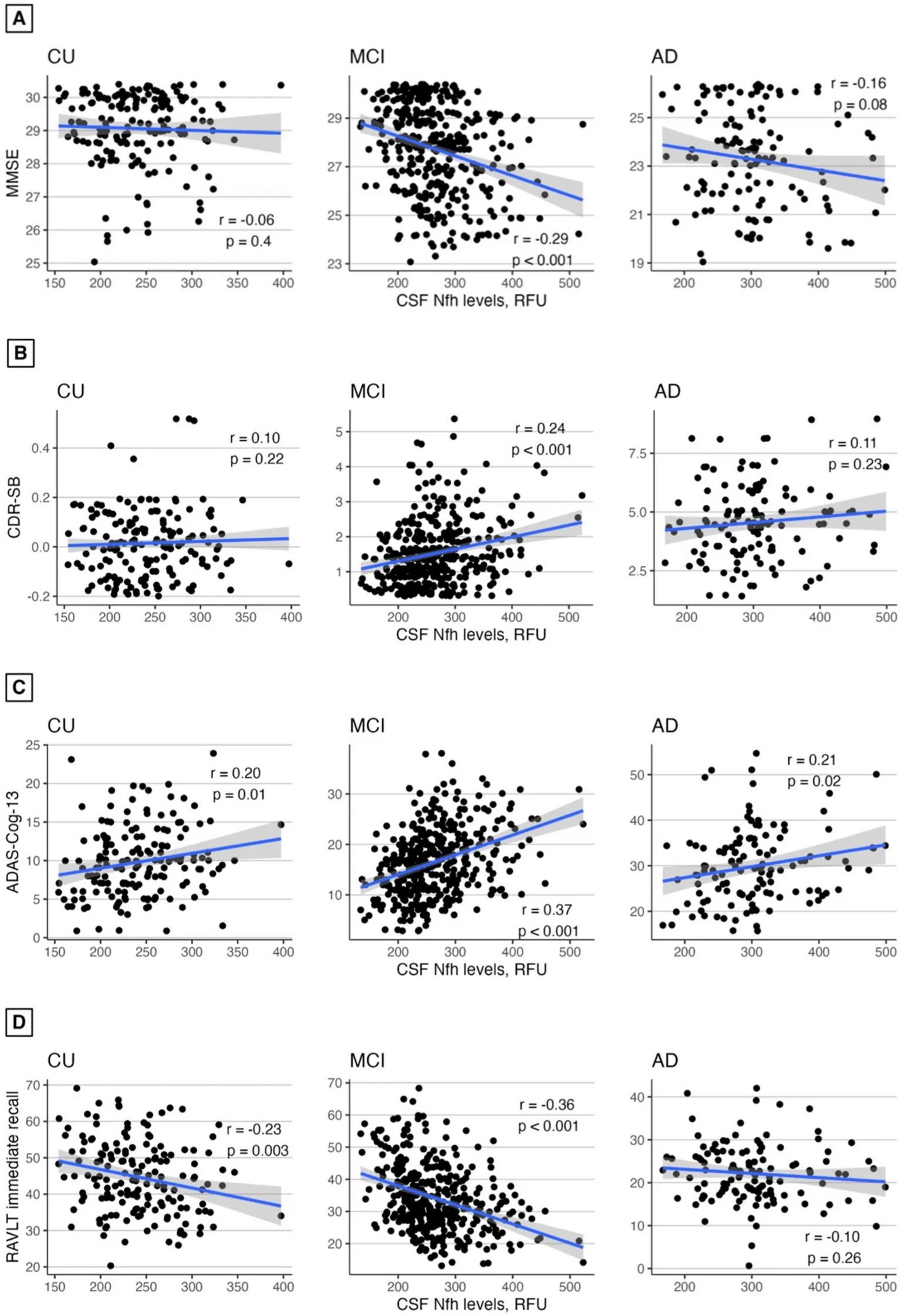
Association of the CSF NfH levels with baseline **(A)** MMSE, **(B)** CDR-SB, **(C)** ADAS-Cog-13, and **(D)** RAVLT immediate recall scores. The correlation results presented in the plots are unadjusted.

Higher CSF NfH levels were significantly associated with poorer cognitive performance in the MCI group, with lower MMSE (*r* = −0.29, *p* < 0.001), higher CDR-SB (*r* = 0.24, *p* < 0.001) and ADAS-Cog-13 (*r* = 0.37, *p* < 0.001), and lower RAVLT immediate recall scores (*r* = −0.36, *p* < 0.001). Among CU participants, higher NfH levels were correlated with higher ADAS-Cog-13 (*r* = 0.02, *p* = 0.01) and lower RAVLT immediate recall scores (*r* = −0.23, *p* = 0.003) but showed no association with MMSE or CDR-SB (Figures 1C,D). In the AD group, a significant association was observed only between NfH levels and ADAS-Cog-13 score (*r* = 0.21, *p* = 0.02) (Figure 1C).

To further test the relationship between CSF NfH and specific cognitive domains, we performed correlation analyses on composite scores for memory, executive function, language and visuospatial functioning (Figure 2). In the MCI group, higher CSF NfH levels significantly were related to poorer performance on composite scores for memory (*r* =−0.41, *p* < 0.001), language (*r* = −0.34, *p* < 0.001), and executive function (*r* = −0.33, *p* < 0.001). Among CU participants, we observed that higher CSF NfH levels were correlated with lower memory (*r* =−0.22, *p* = 0.004), language (*r* = −0.28, *p* < 0.001), and executive function (*r* = −0.17, *p* = 0.03) composite scores. In the AD group, a significant negative correlation was observed only for language domain (*r* = −0.25, *p* = 0.005). No significant result was observed in visuospatial functioning in any group.

**Figure 2:**
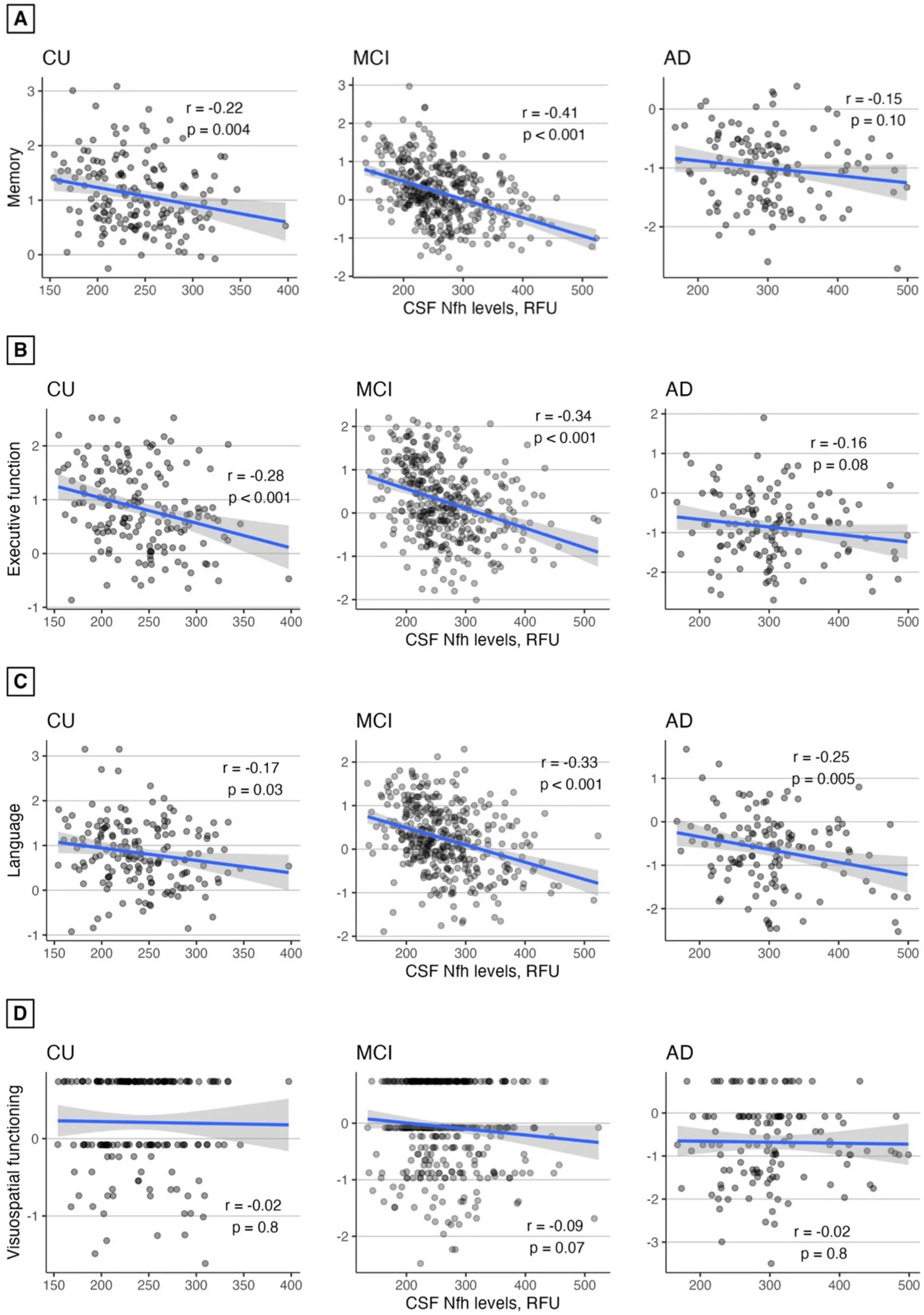
Association of the CSF NfH levels with multiple cognitive composite scores at baseline: **(A)** memory, **(B)** executive function, **(C)** language, and **(D)** visuospatial ability. The correlation results presented in the plots are unadjusted.

To account for potential confounding effects, multivariable linear regression models were performed for testing the cross-sectional associations between baseline CSF NfH and all cognitive outcomes, controlling for age, sex, education, and APOE ε4 status (Supplementary Table S7). In the MCI group, increased baseline CSF NfH levels remained associated with poorer performance across most cognitive outcomes (all *p* < 0.001), except for visuospatial ability. In the AD dementia group, significant associations were observed only for ADAS-Cog-13 (*p* = 0.027) and language (*p* = 0.021). In contrast, in the CU group, the significant associations did not persist after adjusting for confounders (all *p* > 0.05; Supplementary Table S7).

### Longitudinal association of baseline CSF NfH levels with cognitive decline

To investigate the longitudinal associations between baseline NfH levels and cognitive changes (including MMSE, CDR-SB, ADAS-Cog-13 and RAVLT immediate recall scores), linear mixed-effects models were fitted separately for the CU, MCI and AD groups. We also analyzed the four cognitive composite scores as secondary outcomes.

Among CU participants, we found that higher CSF NfH levels were associated with faster decline in MMSE, ADAS-Cog13 scores, executive function, and visuospatial functioning (all *p* < 0.05; Figure 3 and Tables 2, 3). However, these associations did not retain statistical significance after FDR correction (all P_fdr_ > 0.05). In the MCI group, higher CSF NfH levels were significantly associated with faster deline across all cognitive measures (MMSE: *β* = −0.005; CDR-SB: *β* = 0.004; ADAS-Cog-13: *β* = 0.011; RAVLT immediate recall: *β* = −0.007; all *p* < 0.001 and P_fdr_ < 0.001; Figure 3 and Table 2) as well as four composite domains (memory, executive function, language and visuospatial function; all *p* < 0.001 and P_fdr_ ≤ 0.002; Figure 4 and Table 3). No significant results were observed in the AD dementia group.

**Figure 3:**
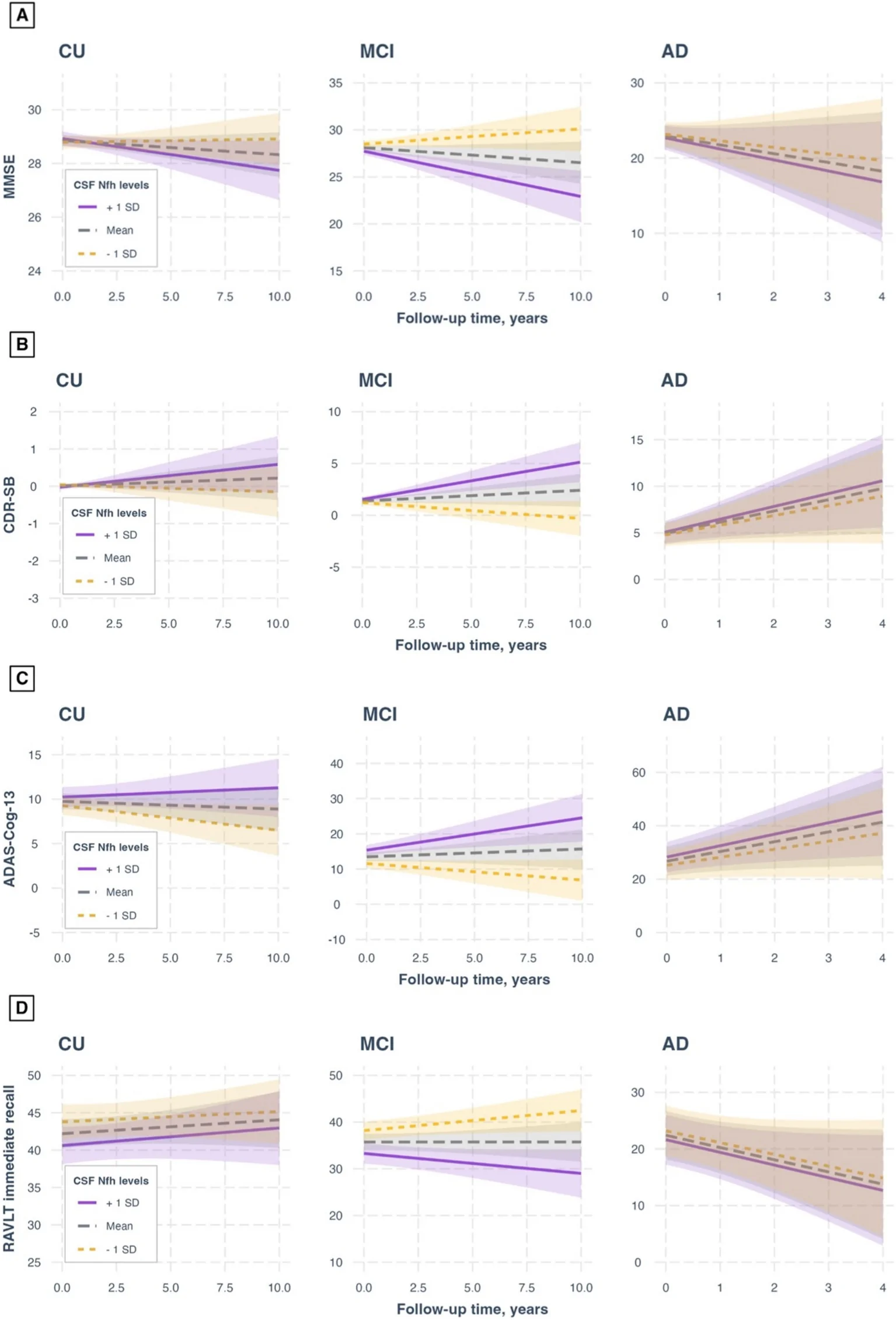
Association of the CSF NfH levels with longitudinal changes of **(A)** MMSE, **(B)** CDR-SB, **(C)** ADAS-Cog-13, and **(D)** RAVLT immediate recall scores.

**Figure 4:**
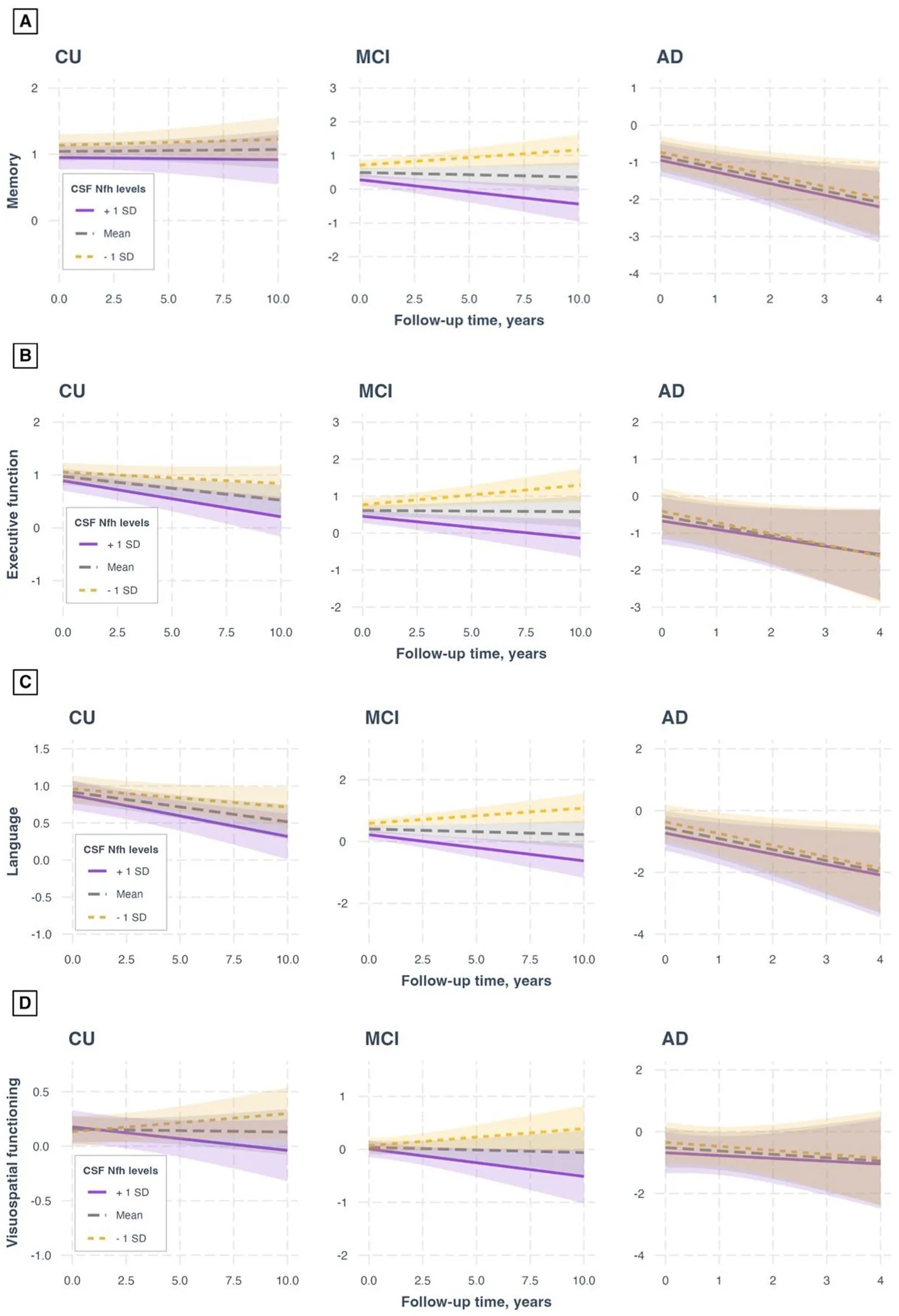
Association of the CSF NfH levels with longitudinal changes of multiple cognitive composite scores: **(A)** memory, **(B)** executive function, **(C)** language, and **(D)** visuospatial ability.

**Table 2:** Summary of linear mixed-effect models with MMSE, CDR-SB, ADAS-Cog-13, and RAVLT total score as outcomes.

Variables	CU	*P*	MCI	*p*	AD	*p*
	Estimate [95% CI]		Estimate [95% CI]		Estimate [95% CI]	
MMSE						
Age × years	−0.010 [−0.021, 0.001]	0.071	0.007 [−0.010, 0.024]	0.409	0.075 [0.016, 0.135]	0.013
Female sex × years	−0.011 [−0.125, 0.104]	0.857	−0.142 [−0.368, 0.083]	0.216	−0.397 [−1.389, 0.596]	0.430
Education × years	−0.006 [−0.027, 0.015]	0.564	−0.001 [−0.040, 0.039]	0.965	−0.039 [−0.210, 0.131]	0.650
APOE ε4 carriers × years	−0.009 [−0.141, 0.122]	0.891	−0.217 [−0.461, 0.026]	0.079	1.574 [0.454, 2.694]	0.006
Aβ + × years	−0.151 [−0.272, −0.030]	0.015	−0.643 [−0.899, −0.386]	<0.001	−2.293 [−4.306, −0.279]	0.026
NfH levels × years	−0.001 [−0.003, −0.000]	**0.050**	−0.005 [−0.007, −0.003]	**<0.001**	−0.004 [−0.011, 0.003]	0.242
CDR-SB						
Age × years	0.000 [−0.007, 0.008]	0.988	0.005 [−0.007, 0.016]	0.439	−0.017 [−0.052, 0.018]	0.339
Female sex × years	0.021 [−0.058, 0.100]	0.601	0.175 [0.016, 0.334]	0.032	−0.002 [−0.585, 0.582]	0.996
Education × years	−0.005 [−0.019, 0.010]	0.526	−0.010 [−0.038, 0.018]	0.472	−0.001 [−0.101, 0.100]	0.988
APOE ε4 carriers × years	0.069 [−0.021, 0.160]	0.133	0.269 [0.099, 0.439]	0.002	−0.768 [−1.427, −0.109]	0.023
Aβ + × years	0.101 [0.017, 0.184]	0.018	0.401 [0.221, 0.581]	<0.001	1.151 [−0.031, 2.333]	0.056
NfH levels × years	0.001 [−0.000, 0.002]	0.079	0.004 [0.003, 0.005]	**<0.001**	0.002 [−0.002, 0.007]	0.254
ADAS-Cog-13						
Age × years	0.001 [−0.030, 0.032]	0.966	−0.019 [−0.059, 0.020]	0.337	−0.203 [−0.316, −0.090]	<0.001
Female sex × years	0.332 [0.008, 0.657]	0.045	0.543 [0.016, 1.071]	0.044	0.663 [−1.252, 2.577]	0.493
Education × years	−0.004 [−0.064, 0.055]	0.882	0.002 [−0.090, 0.094]	0.970	0.062 [−0.272, 0.395]	0.713
APOE ε4 carriers × years	0.468 [0.095, 0.840]	0.014	0.696 [0.127, 1.265]	0.017	−1.473 [−3.612, 0.667]	0.175
Aβ + × years	0.365 [0.024, 0.707]	0.036	1.575 [0.976, 2.173]	<0.001	2.267 [−1.533, 6.066]	0.239
NfH levels × years	0.004 [0.000, 0.008]	**0.042**	0.011 [0.006, 0.016]	**<0.001**	0.009 [−0.005, 0.023]	0.185
RAVLT						
Age × years	−0.018 [−0.066, 0.031]	0.469	−0.006 [−0.038, 0.025]	0.696	0.143 [0.065, 0.222]	<0.001
Female sex × years	−0.193 [−0.697, 0.312]	0.451	−0.446 [−0.857, −0.036]	0.033	−0.102 [−1.356, 1.153]	0.873
Education × years	0.049 [−0.043, 0.140]	0.292	0.023 [−0.049, 0.096]	0.522	−0.042 [−0.261, 0.176]	0.702
APOE ε4 carriers × years	−0.652 [−1.229, −0.076]	0.027	−0.648 [−1.095, −0.202]	0.005	1.297 [−0.253, 2.847]	0.101
Aβ + × years	−0.753 [−1.284, −0.222]	0.006	−0.868 [−1.330, −0.407]	<0.001	−1.719 [−4.316, 0.878]	0.193
CSF NfH levels × years	0.001 [−0.005, 0.008]	0.739	−0.007 [−0.010, −0.003]	**<0.001**	−0.001 [−0.011, 0.008]	0.815

*P*-values in the table represent unadjusted values for the baseline CSF NfH×time interaction terms. *P* values adjusted by FDR (P_fdr_) are as follows: CU group: MMSE: P_fdr_ = 0.100; CDR-SB: P_fdr_ = 0.145; ADAS-Cog-13: P_fdr_ = 0.091; RAVLT immediate recall: P_fdr_ = 0.812. MCI group: MMSE: P_fdr_ < 0.001; CDR-SB: P_fdr_ <0.001; ADAS-Cog-13: P_fdr_ < 0.001; RAVLT immediate recall: P_fdr_ < 0.001. AD dementia group: MMSE: P_fdr_ = 0.358; CDR-SB: P_fdr_ =0.358; ADAS-Cog-13: P_fdr_ = 0.296; RAVLT immediate recall: P_fdr_ = 0.850. FDR correction was applied across all 24 longitudinal models (8 cognitive outcomes across CU, MCI, and AD dementia groups). Bold values indicate *p* < 0.05.

All reported coefficients represent unstandardized β estimates [95% CI] for the interaction between CSF NfH and follow-up time (NfH × time). For example, for MMSE, a β of −0.001 denotes an annual decline of 0.001 point in the MMSE score for every 1-unit increase in NfH levels.

CU, cognitively unimpaired; MCI, mild cognitive impairment; AD, Alzheimer’s disease; APOE, Apolipoprotein E; NfH, neurofilament heavy chain; MMSE, Mini-Mental State Examination; CDR-SB, Clinical Dementia Rating Sum of Boxes; ADAS-Cog-13, Alzheimer's Disease Assessment Scale Cognitive Subscale 13-item version; RAVLT, Rey Auditory Verbal Learning Test; CI, confidence intervals; FDR, alse discovery rate.

**Table 3:** Summary of linear mixed-effect models with multiple cognitive composite scores as outcomes.

Composite scores	CU	*p*	MCI	*p*	AD	*p*
	Estimate [95% CI]		Estimate [95% CI]		Estimate [95% CI]	
Memory						
Age × years	−0.002 [−0.005, 0.002]	0.368	−0.001 [−0.004, 0.002]	0.576	0.011 [0.004, 0.018]	0.002
Female sex × years	−0.022 [−0.059, 0.016]	0.260	−0.044 [−0.082, −0.005]	0.027	−0.001 [−0.115, 0.113]	0.982
Education × years	0.002 [−0.005, 0.009]	0.523	−0.001 [−0.008, 0.005]	0.675	0.004 [−0.016, 0.023]	0.716
APOE ε4 carriers × years	−0.041 [−0.085, 0.002]	0.060	−0.073 [−0.115, −0.031]	<0.001	0.194 [0.064, 0.324]	0.004
Aβ + × years	−0.049 [−0.089, −0.009]	0.016	−0.124 [−0.168, −0.080]	<0.001	−0.199 [−0.428, 0.030]	0.087
NfH levels × years	−0.000 [−0.001, 0.000]	0.578	−0.001 [−0.001, −0.001]	**<0.001**	−0.000 [−0.001, 0.001]	0.912
Executive function						
Age × years	−0.001 [−0.005, 0.002]	0.477	0.002 [−0.001, 0.005]	0.244	0.011 [0.001, 0.020]	0.024
Female sex × years	0.033 [−0.002, 0.068]	0.068	−0.057 [−0.096, −0.018]	0.005	−0.067 [−0.225, 0.091]	0.402
Education × years	0.002 [−0.004, 0.009]	0.498	−0.003 [−0.010, 0.004]	0.401	0.005 [−0.023, 0.032]	0.742
APOE ε4 carriers × years	−0.016 [−0.056, 0.024]	0.423	−0.029 [−0.072, 0.013]	0.174	0.043 [−0.139, 0.224]	0.642
Aβ + × years	−0.017 [−0.054, 0.020]	0.371	−0.116 [−0.160, −0.072]	<0.001	−0.098 [−0.417, 0.221]	0.542
NfH levels × years	−0.001 [−0.001, −0.000]	**0.023**	−0.001 [−0.001, −0.001]	**<0.001**	0.001 [−0.001, 0.002]	0.341
Language						
Age × years	−0.001 [−0.004, 0.002]	0.643	0.001 [−0.002, 0.004]	0.503	0.014 [0.005, 0.023]	0.003
Female sex × years	0.008 [−0.024, 0.039]	0.624	−0.049 [−0.092, −0.006]	0.027	−0.011 [−0.166, 0.144]	0.888
Education × years	0.001 [−0.005, 0.006]	0.850	−0.004 [−0.012, 0.003]	0.269	−0.000 [−0.027, 0.026]	0.978
APOE ε4 carriers × years	−0.017 [−0.052, 0.019]	0.359	−0.055 [−0.102, −0.008]	0.022	0.114 [−0.061, 0.289]	0.200
Aβ + × years	0.003 [−0.030, 0.036]	0.865	−0.104 [−0.153, −0.055]	<0.001	−0.146 [−0.457, 0.165]	0.353
NfH levels × years	−0.000 [−0.001, 0.000]	0.089	−0.001 [−0.001, −0.001]	**<0.001**	0.000 [−0.001, 0.001]	0.634
Visuospatial ability						
Age × years	0.001 [−0.002, 0.004]	0.475	0.002 [−0.002, 0.005]	0.345	0.014 [0.001, 0.027]	0.036
Female sex × years	0.007 [−0.023, 0.037]	0.628	−0.030 [−0.073, 0.014]	0.181	0.032 [−0.182, 0.246]	0.765
Education × years	−0.000 [−0.006, 0.005]	0.884	−0.001 [−0.009, 0.006]	0.717	0.043 [0.007, 0.079]	0.019
APOE ε4 carriers × years	0.002 [−0.032, 0.036]	0.911	−0.013 [−0.061, 0.035]	0.593	0.125 [−0.121, 0.371]	0.319
Aβ + × years	−0.037 [−0.069, −0.005]	0.022	−0.071 [−0.120, −0.022]	0.005	−0.299 [−0.729, 0.131]	0.172
CSF NfH levels × years	−0.000 [−0.001, −0.000]	**0.029**	−0.001 [−0.001, −0.000]	**<0.001**	0.000 [−0.001, 0.002]	0.744

*P*-values in the table represent uncorrected values for the baseline CSF NfH×time interaction terms. P values adjusted by FDR (P_fdr_) are as follows: CU group: ADNI-MEM: P_fdr_ = 0.731; ADNI-EF: P_fdr_ = 0.062; ADNI-LAN: P_fdr_ = 0.153; ADNI-VS: P_fdr_ = 0.069. MCI group: ADNI-MEM: P_fdr_ < 0.001; ADNI-EF: P_fdr_ < 0.001; ADNI-LAN: P_fdr_ < 0.001; ADNI-VS: P_fdr_ = 0.002. AD dementia group: ADNI-MEM: P_fdr_ = 0.912; ADNI-EF: P_fdr_ = 0.454; ADNI-LAN: P_fdr_ = 0.761; ADNI-VS: P_fdr_ = 0.812. FDR correction was applied across all 24 longitudinal models (8 cognitive outcomes across CU, MCI, and AD dementia groups). Bold values indicate *p* < 0.05.

All reported coefficients represent unstandardized β estimates [95% CI] for the interaction between CSF NfH and follow-up time (NfH × time). For example, for MMSE, a β of -0.001 denotes an annual decline of 0.001 point in the MMSE score for every 1-unit increase in NfH levels.

CU, cognitively unimpaired; MCI, mild cognitive impairment; AD, Alzheimer’s disease; APOE: Apolipoprotein E; NfH, neurofilament heavy chain; CI, confidence intervals; FDR, False discovery rate.

The sensitivity analyses restricted to ≤ 5 years of follow-up are concordant with the primary results: all effect directions were consistent, and only three borderline‑significant associations in the CU group did not retain significance (Supplementary Table S6).

### Association of baseline CSF NfH levels with clinical progression

The Kaplan-Meier analysis indicated that CU individuals with high levels of CSF NfH had a significantly higher rate of progression to MCI or AD than the low NfH group (Log-rank test, χ^2^ = 8.8, *p* = 0.003; Figure 5A). Similarly, clinical progression rates differed significantly between the high and low NfH groups among the MCI participants (Log-rank test, χ^2^ = 43.7, *p* < 0.001; Figure 5B). Results for tertile‑based NfH are presented in Supplementary Figure S3.

**Figure 5:**
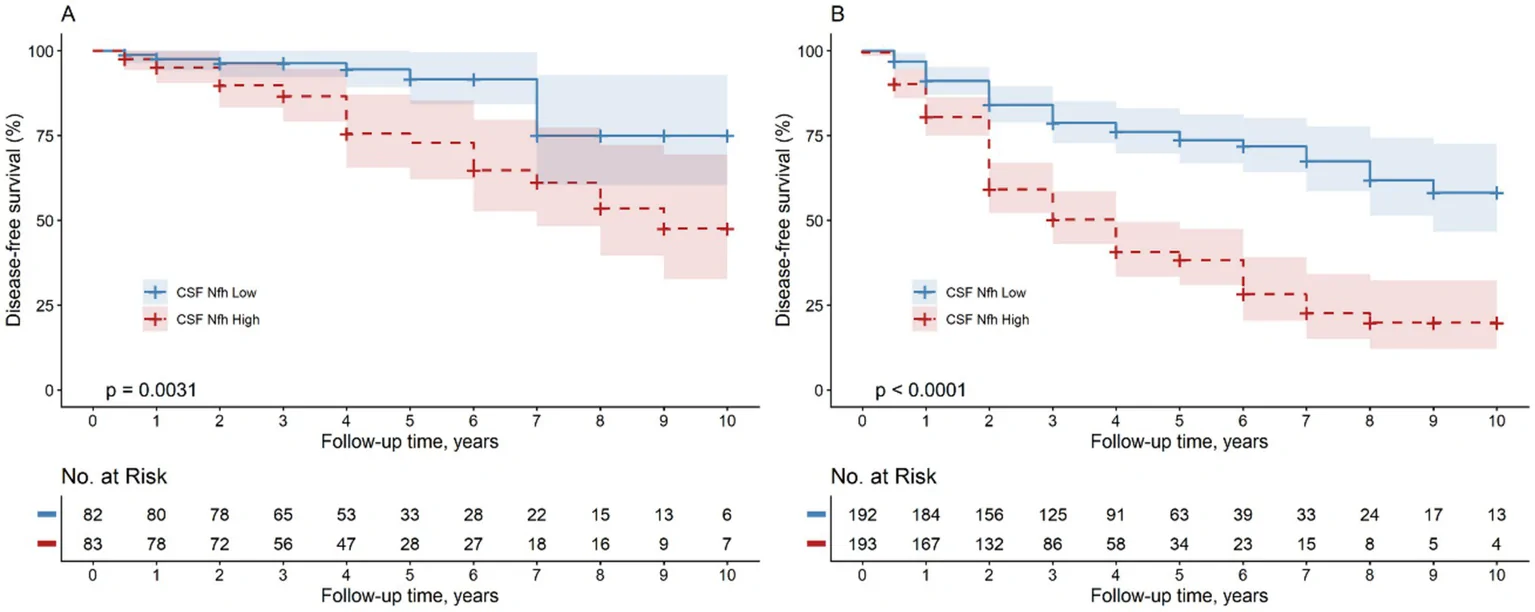
Kaplan-Meier analysis of disease-free probability stratified by baseline CSF NfH levels in participants with CU and MCI. **(A)** Probability of progression from CU to MCI or AD dementia, stratified by low vs. high NfH levels (cut-off at median: 238.4 RFU; Log-rank test, χ^2^ = 8.8, *p* = 0.003). **(B)** Probability of progression from MCI to AD dementia, stratified by low vs. high NfH levels (cut-off at median: 254.4 RFU; Log-rank test, χ^2^ = 43.7, *p* < 0.001). Cross tick marks ("+") represent censored observations. Shaded areas indicate 95% confidence intervals.

Cox proportional hazards models were fitted to further test the association between CSF NfH levels (as a continuous variable) and clinical progression, adjusted for age, sex, education, APOE ε4 status, and amyloid status. Higher baseline NfH levels were significantly associated with an increased risk of clinical progression in both the CU and MCI groups (CU: HR [95% CI] = 1.01 [1.00–1.02], *p* = 0.025; MCI: HR [95% CI] = 1.01 [1.00–1.01], *p* < 0.001; Supplementary Table S2).

### Sensitivity analysis

To further assess whether the observed cognitive associations were specific to NfH or reflected by NfL, we repeated the linear mixed‑effects analyses for CSF NfL in each diagnostic group adjusted for covariates (presented in Supplementary Table S4). In the CU group, CSF NfH was significantly associated with MMSE (*β* = −0.0009, *p* = 0.0499), ADAS‑Cog‑13 (*β* = 0.0008, *p* = 0.0418), and composite scores for executive function (*β* = −0.0006, *p* = 0.0231) and visuospatial ability (*β* = −0.0007, *p* = 0.0288), while NfL was significantly associated with CDR‑SB (*β* = 0.0003, *p* = 0.0071), ADAS‑Cog‑13 (*β* = 0.0002, *p* = 0.0153), and language (*β* = −0.0001, *p* = 0.0094). In the MCI group, CSF NfH was significant for all outcomes, whereas NfL was significant for 7 of 8, except visuospatial ability (*p* = 0.0572; Supplementary Table S4). In contrast, among participants with AD, only NfL demonstrated a significant association with ADAS‑Cog‑13 (*p* = 0.0218). The two CSF biomarkers, NfL and NfH, exhibited distinct associations with cognitive outcomes, suggesting potentially complementary association patterns in the CU and MCI groups. In addition, CSF NfH and NfL were strongly correlated across all diagnostic groups (Spearman *ρ*: CU = 0.71, MCI = 0.76, AD = 0.77; all *p* < 0.001; Supplementary Figure S2).

To contextualize its relevance in the established biomarker framework, we demonstrated the associations between CSF NfH levels and the ATN biomarkers across three diagnostic groups (Supplementary Table S3). To further address the potential confounding effect of amyloid pathology, linear mixed-effects models evaluating the three‑way interaction effects (NfH × time × Aβ status) in four cognitive mearsures were fitted, which was not significant in CU or MCI (all *p* > 0.05; Supplementary Table S5). Furthermore, Aβ-stratified analyses of the association between baseline CSF NfH and cognitive decline are presented in Supplementary Table S8. These results indicate that the predictive value of CSF NfH for cognitive decline is not contingent on amyloid status, supporting its utility as a neurodegeneration biomarker applicable across the MCI spectrum.

## Discussion

In this study, we investigated the relationship between CSF NfH levels and global cognition and multiple cognitive domains across AD spectrum. We found significant cross-sectional correlations between higher levels of CSF NfH and poorer global and domain-specific cognitive performance in participants with MCI, except for visuospatial ability. Longitudinal analyses also revealed higher CSF NfH levels were associated with faster decline in global cognitive decline and in the specific domains of memory, language, executive function and visuospatial ability among MCI participants. Interestingly, this association varied across the clinical spectrum of AD, showing a stage-specific pattern in both CU group and AD groups. Moreover, baseline CSF NfH levels served as a predictor of disease progression along the AD continuum in both CU and MCI groups, supporting the role of CSF NfH as a biomarker of disease progression along the AD continuum.

A key novel finding of our study is that CSF NfH is robustly associated with both baseline cognitive impairment and future decline specifically in individuals with MCI. In the MCI group, higher NfH levels were correlated with poorer cognitive performance across multiple domains at baseline and significantly predicted more rapid cognitive decline overtime. This aligns with the view that MCI represents a highly active phase of neurodegeneration (Wang et al., 2023), during which axonal injury, reflected by elevated NfH levels, is widespread (Khalil et al., 2024).

In the CU group, the associations between baseline CSF NfH levels and cognitive decline observed in certain cognitive measures (MMSE, ADAS-Cog-13, executive function and visuospatial functioning) did not survive multiple testing correction (P_fdr_ > 0.05), suggesting that these findings should be interpreted with caution. Nonetheless, the consistent direction of effects and trend-level associations (P_fdr_ < 0.10) warrant further investigation in larger preclinical cohort. In the AD dementia group, we observed only significant cross-sectional correlation between NfH levels and both the ADAS-Cog-13 score and the language composite score at baseline, while no significant findings were detected in longitudinal analysis. Consistent with previous research, elevated NfH levels have been observed in AD and have also been associated with other neurological disorders, including multiple system atrophy, progressive supranuclear palsy, and amyotrophic lateral sclerosis (de Jong et al., 2007; Petzold et al., 2016; Wang et al., 2022; Khalil et al., 2024).

Neurofilament proteins show promise for assessing disease progression, monitoring clinical trials and therapeutic responses, and determining prognosis across a spectrum of neurological disorders, particularly the light chain (NfL) (Khalil et al., 2018; Gaetani et al., 2019; Turner et al., 2025). Its levels increase in proportion to neuroaxonal injury in biofluids such as CSF and blood (Turner et al., 2025). While most research in AD has focused on NfL (Bridel et al., 2019; Mattsson et al., 2019; Andersson et al., 2020), our study demonstrated the unique value of CSF NfH. Supplementary Table S4 revealed that NfH and NfL had different association patterns with multiple cognitive outcomes. Differential sensitivity of the two markers to regional neurodegenerative processes was observed, especially in CU participants. Thus, CSF NfH might serve as a bridging marker, linking axonal damage to impairment across multiple cognitive domains changes. This study strengthens the evidence for CSF NfH not only as a marker of global neurodegeneration, but also as a quantifiable index of axonal injury that tracks cognitive decline in MCI.

Investigating the association of CSF NfH and cognition across the AD spectrum represents a further novel aspect of this study. Among CU participants, the association of CSF NfH specifically linked to deficits in measures like ADAS-Cog13, RAVLT immediate, and specific domains (memory and executive function). This selective pattern is critical because it reflects that the axonal injury is not diffusion. It suggests that the impact of damage of axons on cognition is specific and may preferentially affect memory and executive domains at the earliest clinical stage of AD. This is consistent with previous studies identifying these domains as more vulnerable to early AD pathology (Petersen et al., 1999; Gibbons et al., 2012). Among AD participants, this association was restricted primarily to global severity of disease and language function.

The predictive validity of baseline NfH for clinical progression in both CU and MCI groups is a key finding, highlighting its ability as a good biomarker to discriminate risk within these groups. This confirms that axonal injury is not merely an epiphenomenon but an active contributor to clinical worsening. This supports the incorporation of NfH into prognostic models for risk stratification in clinical trials and practice, potentially helping to identify individuals at highest short-term risk of progression who may benefit most from targeted interventions (Khalil et al., 2024).

Several limitations warrant consideration. First, CSF NfH levels are measured only at baseline. Further analyses incorporating longitudinal measurements of CSF NfH are needed. Second, the association of NfH levels with global cognition measures and future cognitive decline is correlational and does not represent causality. Third, in the Aβ-stratified analyses (Supplementary Table S8), some subgroups had limited sample sizes, particularly CU Aβ+ (*n* = 63), which may lead to reduced statistical power in the stratified analyses. Moreover, although our sensitivity analyses restricted to ≤ 5 years of follow-up (Supplementary Table S6) were concordant with those of the primary analysis, the possibility of attrition bias due to selective loss to follow-up cannot be fully excluded.

In this study, we explored the role of CSF NfH across the AD continuum. Our findings establish CSF NfH as a robust and consistent predictors of clinical progression in both CU and MCI participants. These results suggest that CSF NfH could serve as a predictive biomarker for cognitive decline, particularly in individuals with MCI who are at high risk of converting to dementia.

## Data Availability

The data used in this study were obtained from the Alzheimer's Disease Neuroimaging Initiative (ADNI) database. The authors are not permitted to distribute these data directly. Requests to access the datasets should be directed to https://adni.loni.usc.edu/.

## Footnotes

1
https://adni.loni.usc.edu/data-samples/adni-data/

## Group member of Alzheimer’s Disease Neuroimaging Initiative

Data used in preparation of this article were obtained from the Alzheimer’s Disease Neuroimaging Initiative (ADNI) database (adni.loni.usc.edu). As such, the investigators within the ADNI contributed to the design and implementation of ADNI and/or provided data but did not participate in analysis or writing of this report. A complete listing of ADNI investigators can be found at: http://adni.loni.usc.edu/wp-content/uploads/how_to_apply/ADNI_Acknowledgement_List.pdf.
