# Supplementary material for: Cerebrospinal fluid neurofilament heavy protein levels associated with domain-specific cognitive decline in Alzheimer’s disease
Source: Front Aging Neurosci. 2026 Oct 22;18:1826215. doi: 10.3389/fnagi.2026.1826215 (PMC13735100; doi:10.3389/fnagi.2026.1826215)
Supplement: Supplementary file 1 [file Data_Sheet_1.DOCX]

**Supplementary Content**

**Table S1. Summary of sample size at follow-up.**

**Table S2. Summary of Cox proportional hazards models**

**Table S3. Cross-sectional correlations between NfH and AT(N) biomarkers**

**Table S4. Longitudinal associations of baseline CSF NfH and NfL with cognitive decline across diagnostic groups**

**Table S5. Interaction effects of baseline CSF NfH levels, Aβ status and follow-up time on cognitive decline in the CU and MCI groups**

**Table S6. Associations between CSF NfH and cognitive measures in the CU and MCI groups (≤5-year follow-up)**

**Table S7. Cross-sectional associations between baseline CSF NfH and cognitive scores adjusted for covariates**

**Table S8. Aβ-stratified analyses in the CU and MCI groups**

**Figure S1. Flowchart.**

**Figure S2. Correlations between CSF NfH levels and NfL.**

**Figure S3. The disease-free probability based on CSF NfH levels (low vs. mid vs. high) in participants with CU and MCI.**

**eMethods**

**Table S1. Summary of sample size at follow-up.**

| **Characteristic** | **Overall** ^1^ | **CU** ^1^ | **MCI** ^1^ | **AD** ^1^ |
| --- | --- | --- | --- | --- |
| years |  |  |  |  |
| 0 | 678 | 165 | 385 | 128 |
| 0.5 | 661 | 161 | 374 | 126 |
| 1 | 652 | 158 | 374 | 120 |
| 2 | 529 | 146 | 313 | 70 |
| 3 | 334 | 66 | 264 | 4 |
| 4 | 284 | 102 | 181 | 1 |
| 5 | 138 | 34 | 104 | 0 |
| 6 | 117 | 52 | 65 | 0 |
| 7 | 85 | 31 | 54 | 0 |
| 8 | 74 | 37 | 37 | 0 |
| 9 | 47 | 22 | 25 | 0 |
| 10 | 44 | 25 | 19 | 0 |
| ^1^n | | | | |

**Table S2. Summary of Cox proportional hazards models**

|  | **CU** | | |  | **MCI** | | |
| --- | --- | --- | --- | --- | --- | --- | --- |
| **Predictors** | **HR** | **95% CI** | ***p*-value** |  | **HR** | **95% CI** | ***p*-value** |
| Age | 1.04 | 0.97, 1.11 | 0.2 |  | 1.00 | 0.98, 1.03 | 0.8 |
| Sex |  |  |  |  |  |  |  |
| Male | — | — |  |  | — | — |  |
| Female | 0.73 | 0.35, 1.54 | 0.4 |  | 1.11 | 0.80, 1.55 | 0.5 |
| Education | 0.86 | 0.75, 0.98 | 0.022 |  | 0.98 | 0.93, 1.04 | 0.5 |
| APOE ε4 status |  |  |  |  |  |  |  |
| non-carriers | — | — |  |  | — | — |  |
| carriers | 1.14 | 0.49, 2.61 | 0.8 |  | 1.44 | 0.99, 2.08 | 0.054 |
| Amyloid status |  |  |  |  |  |  |  |
| Aβ - | — | — |  |  | — | — |  |
| Aβ + | 2.01 | 0.92, 4.41 | 0.082 |  | 2.99 | 1.83, 4.86 | <0.001 |
| CSF NfH levles | 1.01 | 1.00, 1.02 | 0.025 |  | 1.01 | 1.00, 1.01 | <0.001 |
| Notes: Cox proportional hazards models were performed to examine the associations between CSF NfH levles and the risk of conversion from CU to MCI or AD and MCI to AD dementia. Age, education, sex, APOE ε4 status, baseline Amyloid status were included as covariates. Hazard ratios, 95% confidence intervals, and *p* values are reported for all included variables.  Abbreviations: CU, cognitively unimpaired; MCI, mild cognitive impaired; HR: Hazard ratios; CIs: Confidence intervals; APOE4: Apolipoprotein ε4 allele; Aβ: Amyloid; NfH, neurofilament heavy chain. | | | | | | | |

**Table S3. Cross-sectional correlations between NfH and AT(N) biomarkers**

| **AT(N)** | **Biomarker** | **CU** | **MCI** | **AD** |
| --- | --- | --- | --- | --- |
| A | CSF Aβ42 | -0.27 (0.0004) | -0.27 ( <0.0001) | -0.03 (0.73) |
|  | AV45 PET | 0.28 (0.004) | 0.37 ( <0.0001) | 0.11 (0.4) |
| T | CSF p-tau181 | 0.46 ( <0.0001) | 0.44 ( <0.0001) | 0.2 (0.02) |
| (N) | CSF t-tau | 0.44 ( <0.0001) | 0.46 ( <0.0001) | 0.23 (0.009) |
|  | aHV | -0.26 (0.001) | -0.4 ( <0.0001) | -0.23 (0.02) |
|  | FDG PET | -0.22 (0.01) | -0.31 ( <0.0001) | -0.19 (0.07) |

Notes: Values are Pearson's correlation coefficient (*p* values).

**Table S4. Longitudinal associations of baseline CSF NfH and NfL with cognitive decline across diagnostic groups**

|  | | **NfH** | | | **NfL** | | |
| --- | --- | --- | --- | --- | --- | --- | --- |
|  |  | β (95% CI) | β^a^ | P value | β (95% CI) | β^a^ | P value |
| CU | MMSE | **-0.0015 (-0.0029, -0.0000)** | -0.0009 | **0.0499** | -0.0002 (-0.0004, 0.0001) | -0.0001 | 0.2841 |
|  | CDR-SB | 0.0009 (-0.0001, 0.0019) | 0.0010 | 0.0787 | **0.0003 (0.0001, 0.0004)** | 0.0003 | **0.0071** |
|  | ADAS-Cog-13 | **0.0043 (0.0002, 0.0085)** | 0.0008 | **0.0418** | **0.0010 (0.0002, 0.0018)** | 0.0002 | **0.0153** |
|  | RAVLT | 0.0011 (-0.0053, 0.0075) | 0.0001 | 0.7391 | -0.0004 (-0.0017, 0.0008) | 0.0000 | 0.5178 |
|  | *Composite score* | |  |  |  |  |  |
|  | Memory | -0.0001 (-0.0006, 0.0003) | -0.0002 | 0.5784 | -0.0001 (-0.0001, 0.0000) | -0.0001 | 0.2408 |
|  | Executive function | **-0.0005 (-0.0010, -0.0001)** | -0.0006 | **0.0231** | -0.0001 (-0.0002, 0.0000) | -0.0001 | 0.0757 |
|  | Language | -0.0003 (-0.0007, 0.0000) | -0.0005 | 0.0891 | **-0.0001 (-0.0002, -0.0000)** | -0.0001 | **0.0094** |
|  | Visuospatial ability | **-0.0004 (-0.0008, -0.0001)** | -0.0007 | **0.0288** | -0.0001 (-0.0001, 0.0000) | -0.0001 | 0.1322 |
| MCI | MMSE | **-0.0050 (-0.0071, -0.0030)** | -0.0015 | **<0.0001** | **-0.0006 (-0.0009, -0.0003)** | -0.0002 | **0.0002** |
|  | CDR-SB | **0.0039 (0.0024, 0.0053)** | 0.0018 | **<0.0001** | **0.0004 (0.0002, 0.0006)** | 0.0002 | **0.0012** |
|  | ADAS-Cog-13 | **0.0114 (0.0065, 0.0162)** | 0.0011 | **<0.0001** | **0.0013 (0.0005, 0.0021)** | 0.0001 | **0.0011** |
|  | RAVLT | **-0.0068 (-0.0106, -0.0030)** | -0.0006 | **0.0006** | **-0.0007 (-0.0013, -0.0000)** | -0.0001 | **0.0399** |
|  | *Composite score* | |  |  |  |  |  |
|  | Memory | **-0.0009 (-0.0012, -0.0005)** | -0.0008 | **<0.0001** | **-0.0001 (-0.0002, -0.0000)** | -0.0001 | **0.0011** |
|  | Executive function | **-0.0008 (-0.0012, -0.0005)** | -0.0008 | **<0.0001** | **-0.0001 (-0.0002, -0.0000)** | -0.0001 | **0.0010** |
|  | Language | **-0.0011 (-0.0015, -0.0007)** | -0.0011 | **<0.0001** | **-0.0001 (-0.0002, -0.0001)** | -0.0001 | **0.0004** |
|  | Visuospatial ability | **-0.0007 (-0.0011, -0.0003)** | -0.0008 | **0.0011** | -0.0001 (-0.0001, 0.0000) | -0.0001 | 0.0572 |
| AD | MMSE | -0.0046 (-0.0117, 0.0025) | -0.0012 | 0.2088 | -0.0013 (-0.0026, 0.0000) | -0.0003 | 0.0567 |
|  | CDR-SB | 0.0021 (-0.0020, 0.0063) | 0.0008 | 0.3124 | 0.0006 (-0.0001, 0.0014) | 0.0002 | 0.1172 |
|  | ADAS-Cog-13 | 0.0092 (-0.0043, 0.0227) | 0.0009 | 0.1854 | **0.0029 (0.0005, 0.0053)** | 0.0003 | **0.0218** |
|  | RAVLT | -0.0013 (-0.0108, 0.0082) | -0.0002 | 0.7866 | 0.0004 (-0.0014, 0.0022) | 0.0001 | 0.6584 |
|  | *Composite score* | |  |  |  |  |  |
|  | Memory | -0.0000 (-0.0009, 0.0008) | -0.0001 | 0.9312 | 0.0000 (-0.0002, 0.0002) | 0.0000 | 0.9709 |
|  | Executive function | 0.0005 (-0.0005, 0.0016) | 0.0006 | 0.3236 | 0.0001 (-0.0001, 0.0003) | 0.0001 | 0.2681 |
|  | Language | 0.0002 (-0.0009, 0.0013) | 0.0002 | 0.7019 | -0.0000 (-0.0002, 0.0002) | 0.0000 | 0.8715 |
|  | Visuospatial ability | 0.0002 (-0.0014, 0.0018) | 0.0002 | 0.8448 | -0.0001 (-0.0004, 0.0002) | -0.0001 | 0.6592 |

Notes: NfH estimates are reproduced from Tables 2 and 3. Models were adjusted for age, sex, years of education, APOE ε4 carrier status, and CSF Aβ42 levels, with participant-specific random intercepts and random slopes for time.

^a^Standardized β.

Abbreviations: CU: cognitively unimpaired; MCI: mild cognitive impairment; AD: Alzheimer’s disease; APOE: Apolipoprotein E; NfH: neurofilament heavy chain; NfL: neurofilament light chain; MMSE: Mini-Mental State Examination; CDR-SB: Clinical Dementia Rating Sum of Boxes; ADAS-Cog-13: Alzheimer's Disease Assessment Scale Cognitive Subscale 13-item version; RAVLT: Rey Auditory Verbal Learning Test.

**Table S5. Interaction effects of baseline CSF NfH levels, Aβ status and follow-up time on cognitive decline in the CU and MCI groups**

| **Outcome** | **MMSE** | | **CDRSB** | | **ADAS13** | | **RAVLT** | |
| --- | --- | --- | --- | --- | --- | --- | --- | --- |
|  | **Estimate [95% CI]** | ***p*** | **Estimate [95% CI]** | ***p*** | **Estimate [95% CI]** | ***p*** | **Estimate [95% CI]** | ***p*** |
| **CU group** |  |  |  |  |  |  |  |  |
| Years | 1.006 [0.124, 1.889] | 0.026 | -0.060 [-0.656, 0.536] | 0.842 | -0.525 [-2.995, 1.944] | 0.675 | 0.971 [-2.921, 4.863] | 0.622 |
| NfH | 0.001 [-0.004, 0.005] | 0.743 | -0.000 [-0.001, 0.001] | 0.717 | 0.005 [-0.013, 0.023] | 0.565 | -0.028 [-0.068, 0.012] | 0.172 |
| Aβ+ | -0.489 [-1.948, 0.970] | 0.509 | 0.230 [-0.171, 0.631] | 0.259 | -3.146 [-9.334, 3.043] | 0.317 | 5.012 [-8.654, 18.678] | 0.470 |
| NfH ×Aβ+ | 0.002 [-0.004, 0.008] | 0.521 | -0.001 [-0.003, 0.001] | 0.174 | 0.013 [-0.012, 0.038] | 0.300 | -0.019 [-0.074, 0.037] | 0.503 |
| Years × NfH | -0.001 [-0.003, 0.001] | 0.451 | 0.001 [-0.001, 0.002] | 0.336 | 0.001 [-0.004, 0.007] | 0.582 | -0.001 [-0.010, 0.007] | 0.724 |
| Years ×Aβ+ | 0.263 [-0.381, 0.906] | 0.420 | -0.057 [-0.484, 0.370] | 0.792 | -1.153 [-2.946, 0.639] | 0.205 | -2.153 [-4.993, 0.687] | 0.136 |
| Years × NfH ×Aβ+ | -0.002 [-0.004, 0.001] | 0.197 | 0.001 [-0.001, 0.002] | 0.459 | 0.006 [-0.001, 0.014] | 0.090 | 0.006 [-0.006, 0.017] | 0.323 |
| **MCI group** |  |  |  |  |  |  |  |  |
| Years | 0.243 [-1.174, 1.659] | 0.736 | -0.991 [-1.992, 0.010] | 0.052 | -1.004 [-4.368, 2.359] | 0.557 | 1.185 [-1.469, 3.839] | 0.380 |
| NfH | -0.007 [-0.012, -0.001] | 0.012 | 0.002 [-0.002, 0.005] | 0.319 | 0.026 [0.007, 0.045] | 0.008 | -0.050 [-0.078, -0.023] | <0.001 |
| Aβ+ | -0.186 [-1.696, 1.325] | 0.809 | -0.118 [-1.041, 0.806] | 0.802 | 0.486 [-5.211, 6.182] | 0.867 | -8.029 [-16.186, 0.129] | 0.054 |
| NfH ×Aβ+ | -0.001 [-0.007, 0.005] | 0.798 | 0.001 [-0.003, 0.005] | 0.590 | 0.009 [-0.013, 0.031] | 0.440 | 0.015 [-0.016, 0.047] | 0.346 |
| Years × NfH | -0.002 [-0.006, 0.001] | 0.187 | 0.003 [0.001, 0.005] | 0.016 | 0.009 [0.001, 0.017] | 0.027 | -0.003 [-0.009, 0.004] | 0.390 |
| Years ×Aβ+ | 0.300 [-0.708, 1.309] | 0.558 | 0.091 [-0.624, 0.806] | 0.802 | 0.853 [-1.545, 3.252] | 0.484 | 0.506 [-1.362, 2.373] | 0.594 |
| Years × NfH × Aβ+ | -0.004 [-0.008, 0.000] | 0.057 | 0.001 [-0.002, 0.004] | 0.374 | 0.003 [-0.006, 0.012] | 0.539 | -0.006 [-0.013, 0.002] | 0.136 |

Note: All values represent β coefficients with 95% confidence intervals (CI) and *p*-values in linear mixed-effects models. Models were adjusted for covariates (baseline age, sex, years of education, APOE ε4 status). For brevity, the estimates for covariates and their interactions with time are not shown.

**Table S6. Associations between CSF NfH and cognitive outcomes in the CU and MCI groups (≤5-year follow-up)**

| **Outcome** | **CU** | | | **MCI** | |
| --- | --- | --- | --- | --- | --- |
|  | **β** | **p** | **β** | | **p** |
| MMSE | -0.00185 | 0.0361 | -0.00535 | | <0.0001 |
| CDR-SB | 0.00073 | 0.1193 | 0.00400 | | <0.0001 |
| ADAS-Cog-13 | 0.00256 | 0.2617 | 0.01166 | | <0.0001 |
| RAVLT | 0.00165 | 0.7032 | -0.00810 | | 0.0001 |
| ADNI-MEM | -0.00013 | 0.6658 | -0.00096 | | <0.0001 |
| ADNI-EF | -0.00041 | 0.2083 | -0.00099 | | <0.0001 |
| ADNI-LAN | -0.00019 | 0.4795 | -0.00112 | | <0.0001 |
| ADNI-VS | -0.00048 | 0.1687 | -0.00065 | | 0.0031 |

Note. Linear mixed-effects models restricted to follow-up times **≤** 5 years. β coefficients and p-values correspond to the NfH×time interaction term. All mixed-effects models and adjusted covariates are identical to those used in Table 2 and 3.

**Table S7. Cross-sectional associations between baseline CSF NfH and cognitive scores adjusted for covariates**

|  | CU | | MCI | | AD | |
| --- | --- | --- | --- | --- | --- | --- |
| Outcome | Beta (95% CI) | P | Beta (95% CI) | P | Beta (95% CI) | P |
| MMSE | -0.000 (-0.005, 0.004) | 0.864 | -0.007 (-0.010, -0.003) | **<0.001** | -0.004 (-0.010, 0.001) | 0.124 |
| CDR-SB | 0.000 (-0.000, 0.000) | 0.926 | 0.003 (0.002, 0.005) | **<0.001** | 0.003 (-0.002, 0.007) | 0.264 |
| ADAS-Cog-13 | 0.013 (-0.004, 0.030) | 0.147 | 0.033 (0.021, 0.044) | **<0.001** | 0.026 (0.003, 0.048) | **0.027** |
| RAVLT | -0.031 (-0.067, 0.005) | **0.096** | -0.039 (-0.056, -0.021) | **<0.001** | -0.009 (-0.027, 0.009) | 0.328 |
| Memory | -0.002 (-0.004, 0.000) | **0.096** | -0.004 (-0.005, -0.002) | **<0.001** | -0.001 (-0.003, 0.000) | 0.100 |
| Executive function | -0.002 (-0.005, 0.000) | **0.094** | -0.003 (-0.004, -0.001) | **<0.001** | -0.002 (-0.004, 0.000) | 0.095 |
| Language | -0.001 (-0.004, 0.002) | 0.465 | -0.003 (-0.004, -0.002) | **<0.001** | -0.003 (-0.005, -0.000) | **0.021** |
| Visuospatial ability | 0.001 (-0.001, 0.003) | 0.466 | -0.000 (-0.002, 0.001) | 0.606 | -0.001 (-0.004, 0.001) | 0.270 |
| Notes. Adjusted cross-sectional associations correcting for age, sex, education, and APOE 4 status. | | | | | | |

**Table S8. Aβ-stratified analyses in the CU and MCI groups**

| **Outcome** | **MMSE** | | **CDRSB** | | **ADAS13** | | **RAVLT** | |
| --- | --- | --- | --- | --- | --- | --- | --- | --- |
|  | **Estimate [95% CI]** | ***p*** | **Estimate [95% CI]** | ***p*** | **Estimate [95% CI]** | ***p*** | **Estimate [95% CI]** | ***p*** |
| **CU/ Aβ- (n = 63)** | |  |  |  |  |  |  |  |
| Years | 1.124 [0.329, 1.919] | 0.006 | -0.037 [-0.433, 0.359] | 0.853 | -0.288 [-2.661, 2.085] | 0.809 | 0.272 [-3.653, 4.197] | 0.891 |
| NfH | 0.000 [-0.004, 0.004] | 0.961 | -0.000 [-0.001, 0.001] | 0.977 | 0.007 [-0.012, 0.025] | 0.470 | -0.030 [-0.073, 0.014] | 0.178 |
| Years × NfH | -0.001 [-0.002, 0.001] | 0.288 | 0.001 [-0.000, 0.001] | 0.123 | 0.002 [-0.003, 0.006] | 0.479 | -0.003 [-0.010, 0.004] | 0.441 |
| **CU/ Aβ+ (n = 102)** | |  |  |  |  |  |  |  |
| Years | 1.041 [-0.636, 2.719] | 0.219 | -0.148 [-1.430, 1.133] | 0.818 | -2.566 [-7.498, 2.366] | 0.302 | 0.104 [-7.423, 7.632] | 0.978 |
| NfH | 0.004 [-0.001, 0.010] | 0.121 | -0.002 [-0.004, 0.000] | 0.066 | 0.012 [-0.011, 0.036] | 0.305 | -0.034 [-0.082, 0.014] | 0.160 |
| Years × NfH | -0.002 [-0.005, 0.000] | 0.103 | 0.001 [-0.001, 0.004] | 0.290 | 0.008 [-0.000, 0.016] | 0.065 | 0.008 [-0.004, 0.021] | 0.199 |
| **MCI/ Aβ- (n = 123)** | |  |  |  |  |  |  |  |
| Years | 0.087 [-1.039, 1.213] | 0.879 | -1.037 [-1.980, -0.094] | 0.032 | -2.300 [-4.825, 0.226] | 0.074 | 0.212 [-3.349, 3.773] | 0.906 |
| NfH | -0.006 [-0.011, -0.002] | 0.008 | 0.002 [-0.001, 0.005] | 0.190 | 0.024 [0.004, 0.045] | 0.018 | -0.029 [-0.063, 0.004] | 0.082 |
| Years × NfH | -0.001 [-0.003, 0.001] | 0.187 | 0.002 [0.001, 0.004] | **0.004** | 0.003 [-0.002, 0.007] | 0.227 | -0.003 [-0.009, 0.003] | 0.378 |
| **MCI/ Aβ+ (n = 259)** | |  |  |  |  |  |  |  |
| Years | 0.516 [-1.429, 2.461] | 0.602 | -0.712 [-2.046, 0.621] | 0.294 | 1.250 [-3.236, 5.736] | 0.584 | 1.709 [-1.761, 5.179] | 0.332 |
| NfH | -0.007 [-0.011, -0.004] | <0.001 | 0.003 [0.000, 0.005] | 0.034 | 0.035 [0.021, 0.050] | <0.001 | -0.040 [-0.059, -0.021] | <0.001 |
| Years × NfH | -0.006 [-0.009, -0.004] | **<0.001** | 0.004 [0.002, 0.006] | **<0.001** | 0.013 [0.007, 0.020] | **<0.001** | -0.009 [-0.014, -0.004] | **<0.001** |

Note: Linear mixed-effects models were fitted in the CU and MCI groups separately, adjusting for covariates (including baseline age, sex, years of education, APOE ε4 status). For brevity, the estimates for covariates and their interactions with time are not shown.

**Figure S1. Flowchart.**

**Figure S2. Correlations between CSF NfH levels and NfL.**

**Figure S3.** **The disease-free probability based on CSF NfH levels (low vs. mid vs. high) in participants with CU and MCI.** (A) From CU to MCI or AD. (B) From MCI to AD. Cross tick marks ("+") represent censored observations. Shaded areas indicate 95% confidence intervals.
